# Association between PTEN and clinical-pathological features of osteosarcoma

**DOI:** 10.1042/BSR20190954

**Published:** 2019-07-19

**Authors:** Jian Zhou, Xia Xiao, Wanchun Wang, Yingquan Luo

**Affiliations:** 1 Department of Orthopedics, The Second Xiangya Hospital, Central South University, Changsha 410011, Hunan, China; 2 Department of Sports Medicine Research Center, Central South University, Changsha 410008, Hunan, China; 3 Clinical Nursing Teaching and Research Section, The Second Xiangya Hospital, Central South University, Changsha 410011, Hunan, China; 4 Department of Geriatrics, The Second Xiangya Hospital, Central South University, Changsha 410011, Hunan, China

**Keywords:** Bone tumor., PTEN, Clinicopathological features, Meta-analysis, Osteosarcoma, Prognosis

## Abstract

Previous studies indicated the prognostic value of phosphatase and tensin homolog deleted on chromosome ten (PTEN) in osteosarcoma (OS). There was a great degree of inconsistency between these reports. The aim of this meta-analysis was to investigate the clinicopathological features and prognostic role of PTEN positive expression on OS. We searched NCBI PubMed, Embase, Springer, ISI Web of Knowledge, the Cochrane library, China National Knowledge Internet database (CNKI), Wanfang database, Chinese VIP database and Chinese Biological Medical Database (CBM) for relevant papers published before 28 November 2018. The eligibility of all retrieved studies assessing the relationship between PTEN expression and clinicopathological and prognostic outcomes in OS were incorporated. Pooled odds ratio (OR) and 95% confidence intervals (CIs) were used to estimate the outcomes. A total of 13 studies with 580 OS patients were involved to assess the relationship between PTEN expression and clinicopathological features of OS. PTEN positive expression was significantly associated with male (OR = 1.57, 95% CI: 1.03–2.38, *P*=0.035<0.05) and OS high differentiation (OR = 2.33, 95% CI: 1.26–4.29, *P*=0.007<0.05). Additionally, positive expressions of PTEN predict no neoplasm metastasis (OR = 5.69, 95% CI: 3.64–8.90, *P*<0.05). The results of our study showed that positive expression of PTEN may predict higher 5-year survival in OS with the pooled OR of 8.73 (95% CI: 4.18–18.24, *P*<0.05). The results from the present study suggest that positive expression of PTEN is significantly associated with male, high differentiation, no metastasis and high 5-year overall survival rate in OS.

## Introduction

Osteosarcoma (OS), a primary malignant bone tumor, often occurs among adolescents and children [1–3]. The incidence of OS has been increasing year by year [4]. That has caused a serious impact on the health of children and social stability [5]. Prognostic factors mainly include demographics, response to chemotherapy and tumor size, site, stage. However, the prognosis mechanism of OS is not fully understood by us. It is urgently needed to identify the prognostic markers and therapeutic targets of OS [6,7].

Phosphatase and tensin homolog deleted on chromosome ten (PTEN) was first discovered in 1997 [8]. It was the first tumor suppressor gene with tyrosine phosphatase activity. It was named as phosphatase and tensin homolog deleted on chromosome ten because it was located at 10q23 [9]. Phosphatidylinositol 3,4,5-trisphosphate (PIP3) is one of the key factors in the main regulatory pathway of cell growth, which can stimulate cell growth and induce tissue cell apoptosis [10]. PTEN, by removing one of the three phospho-genes of PIP3, regulates the cell growth pathway, causing the cells to self-destruct, thereby causing abnormal cell death [11]. In addition, the tumor suppressor effect of PTEN is also manifested in the regulation of the cell cycle, and PTEN promotes p27Kip1. Binding to the CyclinE/cyclin-dependent kinase 2 (CDK2) complex inhibits CDK2 kinase activity, prevents cells from entering the S phase, and is associated with down-regulation of RB protein phosphorylation levels [12]. Studies have shown that PTEN is down-regulated in various malignant tissues such as glioma, endometrial cancer, lung cancer and prostate cancer [13–15]. Previous studies have assessed the impact of PTEN expression on the prognosis of patients with OS while the results remained conflicting. For instance, Su et al. [16] claimed that positive expression of PTEN is not associated with gender, age, tumor size and metastasis, while Han et al. [17] and Xie et al. [18] reported that expression of PTEN is associated with OS metastasis. In this report, a meta–analysis of all available studies on PTEN expression and OS patients was conducted to investigate its relationship with prognosis of OS.

## Materials and methods

### Search strategy and study selection

We searched NCBI PubMed, Embase, Springer, ISI Web of Knowledge, the Cochrane library, China National Knowledge Internet database (CNKI), Wanfang database, Chinese VIP database and Chinese Biological Medical Database (CBM) for relevant papers published before 28 November 2018. The following terms: (phosphatase and tensin homolog deleted on chromosome ten or PTEN) and (osteogenic tumor or osteosarcoma) were included in the search strategy by two investigators (Jian Zhou and Yingquan Lou) independently. That was checked repeatedly. No language limitations were imposed.

### Inclusion and exclusion criteria

Inclusion criteria: (i) publications were written in Chinese or English. (ii) Original research. (iii) Sufficient information was provided to estimate odds ratio (OR) with corresponding 95% confidence interval (CI). (iv) Pathological diagnosis (gold standard) was used to diagnose OS. (v) PTEN in OS was measured using commercial reagents.

Exclusion criteria: (i) Studies with absence of survival outcome were excluded. (ii) Repeated studies. (iii) Reviews, cell and animal experiments, case reports, correspondences, talks, expert opinions, letters, and editorials without original data were excluded. (iv) There was no cut–off value in the paper. (v) OS was diagnosed without a biopsy.

### Data extraction

The eligibility of all retrieved studies were evaluated by two investigators (Jian Zhou and Xia Xiao). Two investigators (Jian Zhou and Wanchun Wang) extracted the relevant data independently. Extracted databases were then cross–checked between the two authors to rule out any discrepancy. Data regarding the following for each included studies were extracted independently: publication year, first authors’ surname, PTEN assessment methods and the cut–off definition. Corresponding authors were contacted if further information was needed. The study was excluded if no response was received after sending a reminder. The process was described in our previous study [19].

### Assessment of included studies

The quality of included studies were evaluated by Newcastle–Ottawa quality assessment scale (NOS) [20] with three categories (exposure, selection and comparability) and eight items. The quality assessment values ranged from 0 to 9 stars. Studies with score ≥7 stars were included in this meta–analysis.

### Statistical analysis

We calculated OR with corresponding 95% CI to assess the effect of PTEN positive expression on OS. The heterogeneity between the included studies was evaluated using *I^2^* statistics [21]. When there was no significant heterogeneity (*I^2^* ≤ 50%), the fixed–effects model was used [22]; otherwise, a random–effects model was used for the analysis [23]. Moreover, sensitivity analysis was performed by sequentially omitting individual studies to assess the stability of the results. The possibility of publication bias was assessed via visually assessing the symmetry of Egger’s test and Begg’s funnel plots [24]. All the analyses were conducted by STATA 12.0 software (StataCorp LP, College Station, TX, U.S.A.). A two–tailed *P*<0.05 was considered statistically significant.

## Results

### Search results

**Figure 1:**
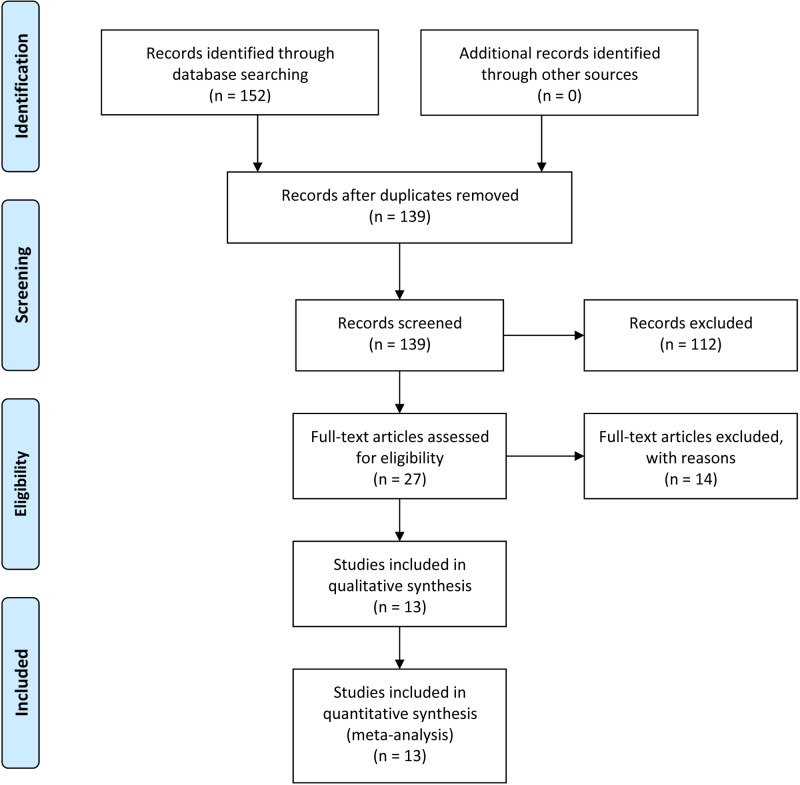
The flow diagram of literature screening

The initial search retrieved 152 articles. Thirteen papers were excluded because of redundant publication. In the remaining 139 articles, 112 manuscripts were excluded because they were not related to the present study. Upon further review, 14 were excluded because they were not clinical studies. Finally, 13 articles [16–18,25–34] published from 2005 to 2017 were adopted in this meta–analysis (Figure 1).

### Study characteristics

As indicated in Table 1, there were 13 studies containing 580 OS patients. The ethnicity involved was only Asian in all eligible articles. Immunohistochemistry (IHC) detection methods were used in all the 13 studies. Among these eligible articles, one study lacked gender and median age. Another paper was not provided the inclusion period.

**Table 1:** Characteristics of studies included in the meta-analysis

First author	Year	Cases	Gender (M/F)	Median age	Inclusion period	Method	NOS score	Reference
Gong et al.	2017	73	NR	NR	2012-2017	IHC	7	[25]
Su et al.	2009	30	19/11	18.00	2004-2006	IHC	8	[16]
Zheng et al.	2009	30	19/12	24.50	2002-2006	SP	7	[26]
Han et al.	2009	35	23/12	20.38	2003-2007	SP	7	[17]
Lei et al.	2009	35	23/12	20.38	2003-2007	SP	8	[27]
Gu et al.	2008	64	38/26	21.40	1996-2001	SP	7	[28]
Xie et al.	2007	40	28/12	19.60	2001-2005	SP	8	[18]
Duan et al.	2007	40	28/12	19.60	2001-2005	SP	8	[29]
Chen et al.	2006	48	27/21	19.00	2000-2005	IHC	7	[30]
Zhong et al.	2006	40	22/18	23.00	1997-2003	SP	7	[31]
Liu et al.	2006	45	30/15	22.50	1990-2002	SP	8	[32]
Song et al.	2005	62	38/24	19.80	NR	SP	8	[33]
Huang et al.	2005	38	21/17	21.40	1995-2002	SP	7	[34]

Abbreviations: NR, no report; SP, streptavidin-perosidase.

### Qualitative assessment

The quality of eligible studies were evaluated using NOS. A higher value (0-9) represents better methodology. The scores of these 13 studies ranging from 7 to 8 (with a mean of 7.46) (Table 1) and detailed information are shown in Supplementary Table S1.

### Association between PTEN expression and OS clinicopathological features

**Figure 2:**
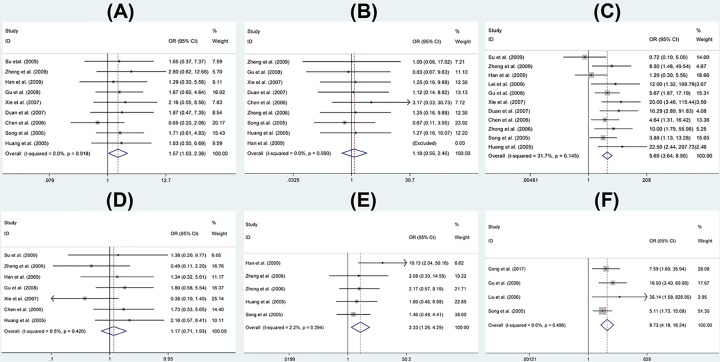
Forest plot showing the association between PTEN expression and OS (**A**) Gender, (**B**) pathological classification, (**C**) metastasis, (**D**) age, (**E**) differentiation, (**F**) overall survival.

In this meta-analysis assessment of correction of PTEN positive expression on OS clinicopathological features, because STATA 12 indicated there was no significant between-study heterogeneity among those eligible studies (*I^2^* < 35%), the fixed-effect model was adopted to detect the pooled OR with corresponding 95% CI. As shown in Figure 2, no statistically significant association between positive expression of PTEN and fibroblastic OS (Figure 2B) or age >25 years old (Figure 2D) was found (*P*>0.05). For the gender and OS differentiation, PTEN positive expression was significantly associated with male (OR = 1.57, 95% CI: 1.03–2.38, *P*=0.035<0.05) (Figure 2A) and OS high differentiation (OR = 2.33, 95% CI: 1.26–4.29, *P*=0.007<0.05) (Figure 2E). Eleven studies were used to assess the association between PTEN positive expression and no metastasis of OS. 4 studies were used to evaluate the relationship between PTEN positive expression and overall survival of OS patients. For no metastasis and 5-year overall survival, the pooled OR of the present study was 5.69 (95% CI: 3.64–8.90, *P*<0.05) (Figure 2C) and 8.73 (95% CI: 4.18–18.24, *P*<0.05) (Figure 2F) respectively, indicating that PTEN expression was significantly associated with no metastasis and overall survival of OS.

### Sensitivity analysis

**Figure 3:**
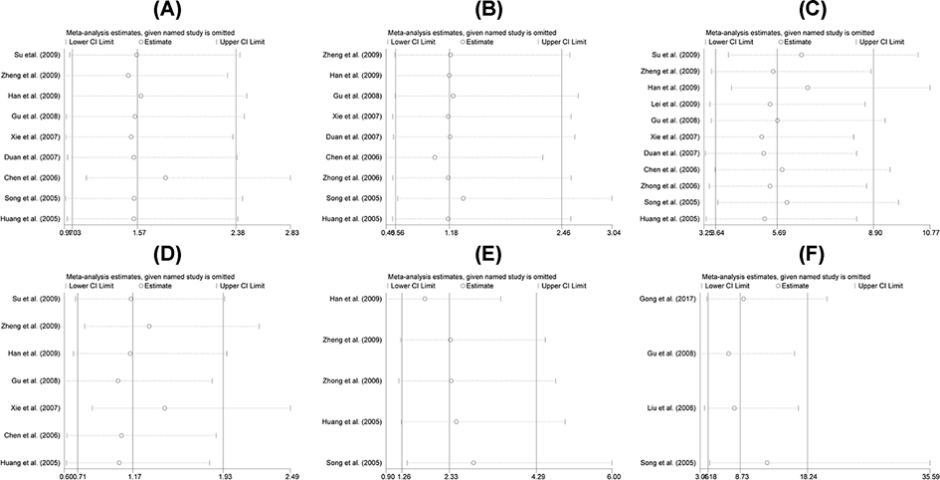
Sensitivity analysis on the association of PTEN expression in OS patients (**A**) Gender, (**B**) pathological classification, (**C**) metastasis, (**D**) age, (**E**) differentiation, (**F**) overall survival.

A sensitivity analysis was conducted to evaluate the stability of the results, which indicated that the combined OR was stable. There was no significant change in heterogeneity when removing a single study. We evaluated the robustness of the results by canceling one study at a time and recalculating the overall OR. A one-time sensitivity analysis was performed to show that our analysis was not too dependent on a study and the conclusion was stable (Figure 3).

### Publication bias

**Figure 4:**
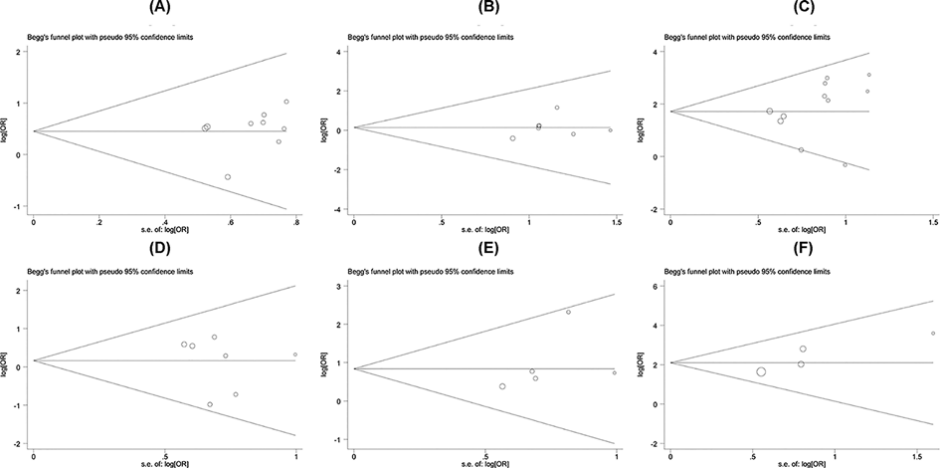
Funnel plot in the meta-analysis of association of PTEN expression in OS patients (**A**) Gender, (**B**) pathological classification, (**C**) metastasis, (**D**) age, (**E**) differentiation, (**F**) overall survival.

Begg’s funnel plot and Egger’s test were conducted to evaluate the publication bias in this meta-analysis. As shown in Figure 4, the funnel plot presented no obvious evidence of asymmetry among the 13 studies. Moreover, no significant publication bias was revealed by Egger’s test in the meta-analysis (*P*>0.05).

## Discussion

OS is the most common malignant bone tumor in adolescents and young adults. OS has the characteristics of easy recurrence and metastasis. Prognosis is poor with low sensitivity to chemotherapy and radiotherapy and treatment for OS has hit the bottleneck. The 10-year survival rate for primary OS patients is only 15% using amputation therapy [35]. OS has a high degree of malignancy, and early metastasis is the main factor affecting whether the patients’ cure rate can be improved. The survival of patients with metastatic OS remains poor. It is urgently needed to update the early prognostic biomarkers to adapt to the appropriate treatment of malignant tumors.

Since PTEN can inhibit cell growth and differentiation, it also promotes apoptosis, making it one of the most interesting tumor suppressor genes after p53. PTEN is down-regulated in a variety of malignancies, and its molecular biology is based on mutations, heterozygous deletions and hypermethylation of the PTEN gene. It has been found that the expression of PTEN in a variety of malignant tumors is closely related to clinical diagnosis and prognosis.

Meta-analysis is a quantitative approach to combine information from different studies on related topics to facilitate the assessment of cancer-related prognostic indicators [36]. A meta-analysis was performed to assess the prognostic role of PTEN positive expression in OS and 13 published articles was included.

The results of this mata-analysis showed that PTEN positive expression was significantly associated with male (OR = 1.57, 95% CI: 1.03–2.38, *P*=0.035<0.05) and OS high differentiation (OR = 2.33, 95% CI: 1.26–4.29, *P*=0.007<0.05). Moreover, PTEN positive expression indicates lower rates of metastasis (OR = 5.69, 95% CI: 3.64–8.90, *P*<0.05) and higher 5-year overall survival (OR = 8.73, 95% CI: 4.18–18.24, *P*<0.05) of OS than PTEN negtive expression. Moreover, a sensitivity analysis was performed to determine the stability of the results. When any single study was removed, the pooled OR was stable with no significant changes. Additionally, no significant publication bias was revealed in the meta-analysis using Egger’s test. PTEN positive expression was significantly associated with OS high differentiation, low metastasis and high overall survival. The reason may be that in addition to promoting cell growth and differentiation, PTEN can also prevent integrin-mediated cell migration by dephosphorylation of focal adhesion kinase (FAK), thereby inhibiting the invasion and metastasis of malignant cells and improving the overall survival rate [37]. In summary, meta-analysis shows that PTEN is a valuable biomarker of OS clinicopathological features.

The limitations of our study needed to be considered. First, no publication bias was found in the selection of documents. Because these studies with desired results are more easily released, which may lead to bias in the overall accuracy, there may still be potential publication bias. Second, although all available data were included in our study, the sample size was still small. Fewer samples may have sample biases and unavoidable random errors in the meta-analysis process, which requires us to conduct a larger sample size study to better assess the relationship between PTEN positive expression and clinicopathological features of OS. Third, although all of the patients we included were pathologically diagnosed. However, the patients may be in different pathological stage, which may have an effect on the result. All that requires a larger sample size and more detailed research program designed to more comprehensive assess the relationship between the two.

## Conclusions

The meta-analysis was conducted to evaluate the relationship between PTEN positive expression and clinicopathological features of OS. Results of our study showed that PTEN was significantly associated with OS high differentiation, low metastasis and high overall survival. However, more well-designed studies with larger sample sizes are still needed to obtain a more comprehensive assessment of the prognostic role of PTEN positive expression and OS.

## Supporting information

**Supplementary Table S1:** Qualitative assessment of included study.

## Abbreviations

CDK2: cyclin-dependent kinase 2
CI: confidence interval
NOS: Newcastle-Ottawa quality assessment scale
OR: odds ratio
OS: osteosarcoma
PIP3: phosphatidylinositol 3,4,5-trisphosphate
PTEN: phosphatase and tensin homolog deleted on chromosome ten
RB: Retinoblastoma
