# Supplementary material for: Association between PTEN and clinical-pathological features of osteosarcoma
Source: Biosci Rep. 2019 Jul 19;39(7):BSR20190954. doi: 10.1042/BSR20190954 (PMC6639452; doi:10.1042/BSR20190954)
Supplement: Supplementary file 1 [file bsr20190954_Supp1.pdf]

Supplementary table 1. Qualitative assessment of included study.

Column	Entries	Study							
		1	2	3	4	5	6	7	8
Section	Is the definition adequate	☆	☆	☆	☆	☆	☆	☆	☆
	Representativeness of the cases	☆	☆	☆	☆	☆	☆	☆	☆
	Selection of controls								
	Definition of controls	☆	☆	☆	☆	☆	☆	☆	☆
Comparability	Comparability of cases and controls on the basis of the design and analysis	☆	☆☆	☆	☆	☆☆	☆	☆☆	☆☆
Exposure	Ascertainment of exposure	☆	☆	☆	☆	☆	☆	☆	☆
	Same method of ascertainment for cases and controls	☆	☆	☆	☆	☆	☆	☆	☆
	Non-Response rate	☆	☆	☆	☆	☆	☆	☆	☆
Total scores		7	8	7	7	8	7	8	8

Notes: 1. Gong et al. 2017, 2. Su et al. 2009, 3. Zheng et al. 2009, 4. Han et al. 2009, 5. Lei et al. 2009, 6. Gu et al. 2008, 7. Xie et al. 2007, 8. Duan et al. 2007.

Column	Entries	Study				
		9	10	11	12	13
Section	Is the definition adequate	☆	☆	☆	☆	☆
	Representativeness of the cases	☆	☆	☆	☆	☆
	Selection of controls					
	Definition of controls	☆	☆	☆	☆	☆
Comparability	Comparability of cases and controls on the basis of the design and analysis	☆	☆	☆☆	☆☆	☆
Exposure	Ascertainment of exposure	☆	☆	☆	☆	☆
	Same method of ascertainment for cases and controls	☆	☆	☆	☆	☆
	Non-Response rate	☆	☆	☆	☆	☆
Total scores		7	7	8	8	7

Notes: 9. Chen et al. 2006, 10. Zhong et al. 2006, 11. Liu et al. 2006, 12. Song et al. 2005, 13. Huang et al. 2005.
